# Ankyloglossia and its surgical correction by frenuloplasty in a she-camel calf (*Camelus dromedarius*)

**Published:** 2012-04-28

**Authors:** S. Anwar, G.N. Purohit

**Affiliations:** 1 *Al Qattara Veterinary Hospital, Al Ain, UAE*; 2 *Department of Veterinary Gynecology and Obstetrics, College of Veterinary and Animal Science, Rajasthan University of Veterinary and Animal Sciences, Bikaner, Rajasthan, India*

**Keywords:** Ankyloglossia, Camel, Frenuloplasty

## Abstract

A rare case of ankyloglossia in a one-humped camel and its successful surgical correction by horizontal to vertical frenuloplasty is reported. Seven-month-old she-camel calf, with a history of excessive salivation and inability to chew and swallow hay, was referred to Al Qattara veterinary hospital. Solid food was regurgitated, whereas milk and water could be swallowed. On examination; the animal could not protrude its tongue which was attached by a thin tissue band between the ventral surface of the tongue and the floor of the oral cavity. The tissue band was corrected by frenuloplasty and the incisions were sutured. The animal recovered well as the salivation and regurgitation stopped and the animal became able to chew and swallow solid food including hay. It was concluded that ankyloglossia can occur in one-humped camel in which a horizontal to vertical frenuloplasty may improve full function of tongue movement.

## Introduction

Ankyloglossia, commonly known as tongue-tie, is a rare congenital oral anomaly that has been reported in dogs (Temizsoylu and Avki, 2003; Kilic and Sari, 2004; Grundmann and Hofmann, 2006; Karahan and Kul, 2009). The condition has been rarely reported in goat kids (Nair and Bandopadyay, 1994) and calves (Mouli, 1993; Orhan *et al.*, 2001; Kilic, 2011). In humans ankyloglossia is a common anomaly in newborn infants (Wright, 1995; Jones and Derrick, 1998; Messner and Lalakea, 2000; Heller *et al.*, 2005). A complete attachment of the lingual frenulum to the floor of the oral cavity leads to limitation of tongue movement and difficulties in eating and swallowing.

Frenuloplasty is the usual surgery suggested for correcting the condition (Temizsoylu and Avki, 2003; Grundmann and Hofmann, 2006; Kilic, 2011). Ankyloglossia has been reported in camels (Ramadan, 1994). This report describes ankyloglossia and its surgical correction in a calf of one-humped camel.

## Case history

A seven-month-old she-camel calf was referred to Al Qattara veterinary hospital with complaints of inability to swallow dry food after chewing. The calf was raised on milk by bottle feeding since its birth due to the death of the dam soon after delivery. The owner noticed that the calf had difficulty in chewing hay, but water and milk could be swallowed. Attempts to consume dry hay by the calf resulted in its regurgitation and throwing feed from mouth. Physical examination revealed that the animal was unable to protrude its tongue. The calf showed signs of weakness and retarded growth, with a continuous drooling of saliva and regurgitation of food.

**Fig. 1:**
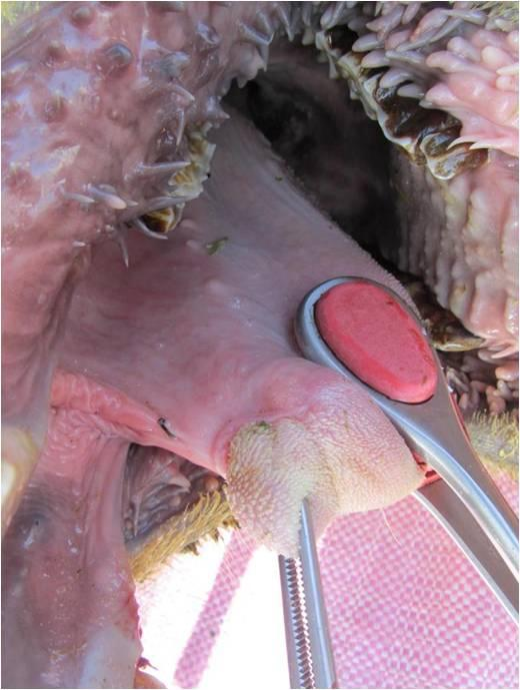
Lateral view of oral cavity with ankyloglossia.

The tip of the tongue was ventrally deviated. A thin tissue band between the ventral surface of the tongue and the floor of the oral cavity was seen when the tongue was pulled out. This tissue band was attaching the tip of the tongue to the ventral surface of the oral cavity (Fig. 1). The oral cavity was free of any other lesions. Frenuloplasty was performed for the correction of ankyloglossia.

## Surgical technique

Horizontal to vertical frenuloplasty was done to correct the problem. The animal was kept off food for 24 hrs. Water was withheld for 12 hours prior to surgery. The animal was sedated with xylazine (0.15 mg/kg) and ketamine (2.5 mg/kg) administered intravenously and kept in lateral recumbency with the mouth opened manually by attendants.

The tip of the tongue was pulled out and kept dorsally by holding it with uterine forceps. The tense tissue band at this moment was surgically incised beginning at the tip of the tongue near to its attachment with the floor of oral cavity. Horizontal incision was extended posteriorly dividing the band between the ventral surface of the tongue and the floor of the oral cavity through the frenulum, and the tongue was sufficiently free to protrude beyond the lower incisors.

**Fig. 2:**
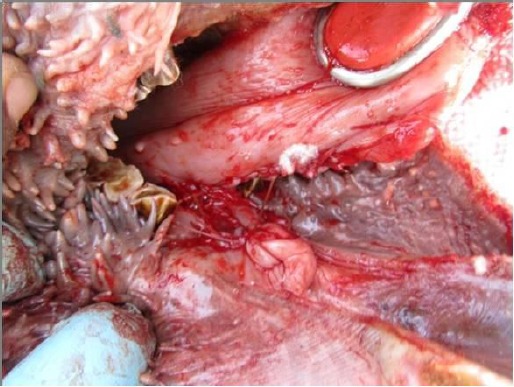
Lateral view of oral cavity with ankyloglossia after horizontal to vertical frenuloplasty.

Care was taken not to injure the frenulum and sub lingual salivary gland ducts. Minor bleeding was controlled by ligation and sponges soaked in epinephrine. The incision was closed at the ventral surface of the tongue and dorsally at the floor of the oral cavity by horizontal mattress sutures using chromic catgut size 2/0 (Fig. 2).

## Postoperative care

Postoperative care included the administration of flunixin meglumine (Finadyne, Schering-Plough, Germany) at a dose of 2 ml/45 kg as a single injection intravenously daily for 3 days, to control pain. Penicillin and Streptomycin suspension (Pen & Strep, Norbrook, UK) was administrated in a dose of 1 ml/kg for 3 days intramuscularly, to prevent infection. The sutures were not removed.

## Outcome

First day following surgery, greater mobility of the tongue was evident. There was an uneventful recovery and drooling of saliva was no more evident. On the follow up, and after a month, it was found that the animal was able to browse and swallow hay. The animal gained weight and had no difficulties in rumination or eating.

## Discussion

Ankyloglossia is a general term that describes a group of congenital anomalies characterized by limitation of tongue movement (Messner and Lalakea, 2000).

In humans, attachment of the tongue tip to the hard palate is referred to as superior ankyloglossia, whereas attachment of the tongue to the lingual frenulum is referred to as inferior ankyloglossia (Wright, 1995). Ankyloglossia is considered as a congenital anomaly of the tongue (Naimer *et al.*, 2003; Hooda *et al.*, 2010) that is usually characterized by a short and thick lingual frenulum. The genetic mutations such as in TBox genes and other fetal mechanism have still been under investigation as possible causes of ankyloglossia (Karahan and Kul, 2009).

Ankyloglossia is rarely combined with other anomalies in human, such as cleft palate, blepharophimosis or microstoma (Warkany, 1975).

In the camel, Ramadan (1994) described similar findings under the term “curling of the tongue”. This defect was described to be due to shortening of frenulum linguae.

In a goat with partial ankyloglossia a thyroglossal cyst under the base of the tongue was observed (Nair and Bandopadyay, 1994).

Most of the clinical signs in ankyloglossia are due to limitation of tongue movements, including difficulties during eating, swallowing and in maintaining the oral hygiene (Ramadan 1994; Kilic and Sari, 2004; Kilic, 2011).

In the present report, the abnormalities in the camel calf were inability to protrude the tongue, drooling of saliva and regurgitation of food with resultant inability of proper feed consumption by the animal. Drooling of saliva may have been related to abnormalities of the swallowing mechanism and has been reported with partial and complete inferior ankyloglossia (Kilic and Sari, 2004; Kilic, 2011).

Partial ankyloglossia was identified in a dog because of inability to protrude the tongue sufficiently to allow passage of an endotracheal tube (Wolff, 1980). For this reason, the calf was not intubated and injectable drugs were used to maintain anesthesia in the camel calf described in this report.

In veterinary medicine, V-shaped and horizontal to vertical techniques have been described (Wolff, 1980; Kilic and Sari, 2004; Kilic, 2011). In the V-shaped frenuloplasty the incision begins near the tip of the tongue where the frenulum is fused and continued caudally along the ventral surface of the tongue. A second incision is made along the floor of the oral cavity (Kilic and Sari, 2004). In this report the horizontal to vertical frenuloplasty as suggested previously (Kilic and Sari, 2004) was used with a good clinical outcome.

In conclusion we report a congenital ankyloglossia in one-humped camel, in which a horizontal to vertical frenuloplasty may improve full function of tongue movement.
